# Sexual and reproductive complications and concerns of survivors of childhood, adolescent and adult cancer

**DOI:** 10.1007/s11764-023-01349-6

**Published:** 2023-03-29

**Authors:** Brigitte Gerstl, Christina Signorelli, Claire E. Wakefield, Rebecca Deans, Tejnei Vaishnav, Karen Johnston, Kristen Neville, Richard J. Cohn, Antoinette Anazodo

**Affiliations:** 1 Kids Cancer Centre, Sydney Childrens Hospital, Randwick, Sydney, NSW 2031 Australia; 2 Behavioural Sciences Unit, Kids Cancer Centre, Sydney Childrens Hospital, Sydney, NSW Australia; 3 School of Clinical Medicine, UNSW Medicine & Health, Randwick Clinical Campus, Discipline of Paediatrics, UNSW , Sydney, Australia; 4 Department of Gynaecology, The Royal Hospital for Women, Sydney, NSW Australia; 5 Fertility Research Centre, The Royal Hospital for Women, Sydney, NSW Australia; 6 Department of Endocrinology, Sydney Children’s Hospital, Sydney, NSW Australia; 7 Nelune Comprehensive Cancer Centre, Prince of Wales Hospital, Sydney, NSW Australia

**Keywords:** Cancer, Oncofertility sexual health, Reproductive health, Survivorship, Patient-reported outcomes

## Abstract

**Purpose:**

Cancer survivors may experience infertility and sexual dysfunction following cancer treatment. Survivors report significant gaps in oncofertility care and consider these issues important, yet they are rarely discussed. The aims of this study were to evaluate survivors’ sexual and reproductive complications across age groups and to identify specific groups of survivors at risk for sexual and reproductive complications.

**Method:**

We report data collected from survivors of cancers diagnosed in childhood, adolescence and adulthood following the development and piloting of a reproductive survivorship patient reported outcome measure (RS-PROM).

**Results:**

One hundred and fifty survivors participated in the study (mean age at cancer diagnosis was 23.2 years [*SD*, 10.3 years]). About 68% of participants expressed concerns about their sexual health and function. Survivors (50%) expressed at least one body image concern, with the female gender the most common risk factor for all subgroups. A total of 36% of participants reported at least one concern regarding their fertility, with more male than female survivors reporting fertility preservation prior to treatment. Females compared with male participants were more likely to feel less physically attractive after treatment (*OR* = 3.83, 95% *CI* = 1.84–7.95, *p* < 0.001). More females than males were also more likely to feel dissatisfied with the appearance of a scar(s) after treatment (*OR* = 2.36, 95% *CI* = 1.13–4.91, *p* = 0.02).

**Conclusion:**

The RS-PROM identified multiple reproductive complications and concerns for cancer survivors in the survivorship period.

**Implications for Cancer Survivors:**

Utilising the RS-PROM in conjunction with a clinic appointment could help identify and address cancer patients’ concerns and symptoms.

**Supplementary Information:**

The online version contains supplementary material available at 10.1007/s11764-023-01349-6.

## Introduction

Child and adolescent cancer survivors may experience a number of adverse reproductive late effects of cancer treatment such as sexual and psychosexual dysfunction [1], endocrine complications [2] and infertility [3]. These complications can have an impact on both physical and psychological wellbeing [4–6] and may impact a patients’ ability to develop and sustain intimate relationships [7]. There are significant gaps in the literature surrounding models of reproductive and sexual healthcare for cancer survivors. Many survivors have difficulties with disclosing reproductive complications or concerns and encounter challenges having discussions with healthcare professionals (HCPs) who can also find these conversations embarrassing or do not have the expertise in this area [8–10]. Moreover, discussions about patients’ sexual and reproductive unmet needs and experiences following cancer treatment can be uncomfortable for patients to broach with their HCPs and are often poorly addressed [8]. The literature has reported outcomes associated with treatment-related infertility after cancer; however, few studies have reported on the sexual and reproductive complications and concerns of cancer patients in the survivorship period [8, 9]. Cancer patients have a strong desire to address topics associated with their sexual and reproductive needs and how these needs may affect potential parenthood and sexual relationships after cancer [10, 11]. Despite numerous national and international oncofertility guidelines [38–40] recommending that cancer patients be referred for consultation with a reproductive specialist, prior to starting cancer treatment as well as in the survivorship period, discussions around reproductive late effects of cancer treatment remain low. The literature reports that patients are not routinely referred for a survivorship consultation, for discussions around sexual and reproductive concerns after successful curative cancer treatment, even though cancer patients have a desire to raise these concerns [6, 8, 41]. Furthermore, reproductive discussions are often poorly documented in patients’ medical notes and reproductive survivorship follow-up remains low [6, 8, 41].

We previously reported on the development, acceptability, feasibility and appropriateness of a reproductive-patient reported outcome measure (RS-PROM) [3], developed to address some of the existing gaps in reproductive survivorship care with the goals of enhancing patient-centred reproductive care and satisfaction and identifying reproductive symptoms and concerns that can be managed in survivorship consultations [8]. To date, there has not been reproductive survivorship patient-reported outcome measure (RS-PROM) integrated into survivorship care. In this study, we report on the data collected from the implementation of the RS-PROM into clinical care in a reproductive survivorship clinic. This study aimed:

- To evaluate survivors’ sexual and reproductive complications across age groups, and
- To identify specific groups of survivors at risk (e.g. gender/age/clinical history) for sexual and reproductive complications and concerns.

## Methods

The RS-PROM had been previously developed and shown to be an acceptable and feasible tool for use in a survivorship clinic [3, 12]. The RS-PROM tool included a series of validated measures to explore clinical, psychosocial and psychosexual concerns that may affect cancer survivors. Additional questions were developed and included to assess pubertal status, hormone function, contraception use and family planning [3]. Text boxes were available for participants to provide additional information relative to specific topics or themes. Cancer survivors completed the RS-PROM based on their own reproductive health experiences.

### Instruments

Based on a systematic review of the literature, the RS-PROM incorporated existing validated measures addressing reproductive health concerns of cancer survivors (Supplementary Figs. II and III) [3, 5, 8, 12, 13]. A series of questions were also developed by the research team to assess puberty, hormonal function, contraception, fertility and future pregnancy.

Basic demographic and clinical information were included in the RS-PROM. Patient sociodemographic information included name, date of birth, gender and sexual orientation. The Kinsey Scale [12, 14] (as well as the Sexuality Scale) was used to describe an individual’s sexual orientation based on the respondent’s responses at a particular time. There are four options included on this scale: heterosexual, bisexual, gay/lesbian and other. The research team added an additional option which included “I prefer not to answer”. Cancer-related characteristics included age at diagnosis, type of cancer and treatment(s) received.

After cancer treatment, the Body Image Scale (BIS) [12, 15] offered an opportunity to better understand a patient’s reproductive concerns. The BIS consisted of ten items, each scored on a Likert Scale of one to five. Poor body image was indicated by lower scores.

Cancer patients were evaluated for their sexual function using the EORTC Sexual Health Questionnaire (EORTC-SHQ-C22) [12, 16–18]. The multidimensional quality of life instrument includes 22 items exploring sexual function and psychosexual questions. Items on sexual fulfilment, sexual pain and single items representing an integrative approach were also included. A higher score on the functioning scales indicated that an individual was functioning better, while a higher score on the symptom scales implied that an individual was experiencing greater severity.

Reproductive Concerns After Cancer (RCAC) Scale [12, 18] consisted of 18 items, each divided into six subscales, each of which included three items. The tool measured fertility potential, fertility disclosure by the partner, child’s health, personal health as well as acceptance of possible infertility and pregnancy. Items were scored using a five-point Likert Scale. Lower scores indicated a stronger agreement with the item.

The RS-PROM also included a section on pubertal development that included ten questions focused on how cancer diagnosis or treatment affected an individual’s pubertal development. With the Emotion Thermometer tool, responding levels for depression, anxiety, distress, anger and need for help were also assessed based on the visual scale of a ‘thermometer’, with higher scores indicating a greater impact in relation to the feeling [12, 18, 19].

### Inclusion criteria

In order to participate in the study, participants had to be > 18 years and ≤ 45 years of age at the time of the study and had completed cancer treatment > 5 years ago. Patients were recruited either directly by a HCP at the Sydney Children’s Hospital paediatric and adolescent and young adult (AYA) long-term survivorship clinic or they were recruited through the Australasian Oncofertility Registry (AOFR) [20], where registered patients are able to indicate their interest in participating in further oncofertility studies.

### Exclusion criteria

Patients were excluded if a treating clinician did not feel that the patient was an appropriate candidate to participate or if the patient did not meet the study’s inclusion criteria.

### Statistical analysis

Descriptive statistics included frequencies and proportions for categorical variables and means and standard deviations (*SD*) for continuous variables. Groups were compared using the Chi-squared (*X*^2^) test or Fisher’s exact test for categorical variables. We also conducted multiple regression analyses to explore demographic and clinical factors associated with sexual and reproductive patient concerns. Variables used in the model included age group at cancer diagnosis [paediatric patients: diagnosed with cancer ≤ 14 years; adolescent and young adult [AYA] patients: diagnosed with cancer 15–25 years (in Australian clinical settings, AYA is usually considered to be 15–25 years of age) and adult patients: diagnosed with cancer ≥ 26 years], gender (male versus female), relationship status at the time of completing the questionnaire (yes versus no), cancer type at diagnosis (blood cancers versus solid tumours), having had fertility preservation (FP) prior to starting cancer treatment (yes versus no) and whether the participant had ever been pregnant after cancer (yes versus no). A two-tailed test with a 5% level of significance was used for all statistical analyses. We analysed data in STATA 14.2 (StataCorp, College Station, TX, USA)[20].

### Reliability

Internal consistency, for each of the measures, was calculated using Cronbach’s alpha (α) reliability coefficient, with a minimum value of 0.70 for retaining items. We used the following criteria to determine levels of reliability: (a) less than 0.50, low reliability; (b) between 0.50 and 0.80, a moderate level of reliability; and (c) greater than 0.80, high reliability [20].

### Ethics approval

We obtained ethics approval from the Sydney Children’s Hospitals Network Human Research Ethics Committee (reference LNR/16/SCHN/396).

## Results

### Demographic information

Of the 214 eligible participants who were contacted to participate in this study, 150 completed the RS-PROM (70.1%); 38.7% (*n* = 58) were male and 61.3% (*n* = 92) were female participants. The mean age at cancer diagnosis for all participants was 23.2 years (*SD*, 10.3 years) [mean age of male survivors: 24.5 years (*SD*, 8.4 years); mean age of female survivors at cancer diagnosis: 22.5 years (*SD*, 11.4 years)]. The mean age at completing the RS-PROM was 29.4 years (range: 19–45 years). The mean follow-up period for the cohort, from cancer diagnosis to completion of the RS-PROM was 23 years (range: 5–45 years).

**Fig. 1:**
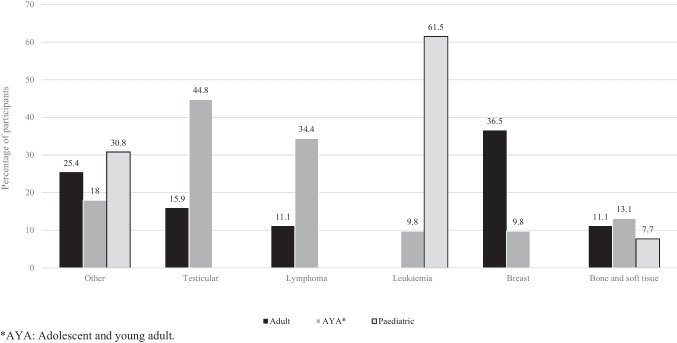
Adult, AYA and paediatric patients diagnosed with cancer (*n* = 150)

Twenty-six (17.3%) participants were diagnosed as a paediatric cancer patient (≤ 14 years), 61 (40.7%) were diagnosed as an AYA patient (15–25 years) and 63 (42%) had been diagnosed as an adult (≥ 26 years). Most participants reported having a previous diagnosis of breast cancer (*n* = 29, 19.3%) or lymphoma (*n* = 28, 18.7%), followed by leukaemia (*n* = 22, 14.7%), testicular (*n* = 19, 12.7%), bone or soft tissue (*n* = 17, 11.3%) and other (*n* = 35, 23.3%) cancers (Fig. 1).

Participants more commonly reported (64.7%) that they were in a relationship and defined themselves as heterosexual (*n* = 137, 91.3%), followed by bisexual (*n* = 4, 2.7%) and homosexual (*n* = 2, 1.3%). Three participants declined to answer this question (*n* = 3, 2%). More male than female participants reported having undergone FP prior to starting cancer treatment (males: 74.1% versus females: 42.4%, *p* < 0.001).

### Puberty

More female than male participants reported having received cancer treatment before starting pubarche (females 31.1% versus males: 11.7%, *p* = 0.01). A total of 88.9% of women had menstruated before starting treatment, with 78.1% indicating that their menstrual cycle had resumed following the end of their cancer treatment; of those who resumed menstruation after cancer treatment, more than half indicated that they had a regular menstrual cycle of between 25 and 36 days (65.2%).

### Hormone treatment and bone health

When participants were asked whether they had been investigated for hormonal problems after completion of cancer treatment, significantly more females compared with male participants had undergone hormone investigations (females: 38.9% versus males: 16.7%, *p* = 0.004). Furthermore, significantly more females compared with male participants stated that they were on hormone therapy after cancer treatment (females: 34.4% versus males: 8.3%, *p* < 0.001).

While we did not have information on the reasons for starting HRT, we did include information on the complications to bones as a result of hormonal changes. Female participants were more likely to have undergone a bone density scan (females: 35.6% versus males: 11.7%, *p* = 0.001) and tended to report higher rates of osteoporosis, brittle, weak or fragile bones (females: 12.2% versus males: 3.3%, *p* = 0.06) but with no difference in fracture rate (females: 15.6% versus males: 10%, *p* = 0.33).

### *Sexual Health and Function Questionnaire* [17, 18]

Most AYA and adult participants stated that they were sexually active prior to starting cancer treatment (93.6%) (AYA: 90.2% and adults 96.8%). A large majority of participants expressed the importance of being sexually active after cancer (85.3%) (females: 80% versus males: 93.3%, *p* = 0.02), with most (76.7%) stating that they were currently sexually active (females: 70% versus males: 86.7%, *p* = 0.02). Most participants revealed that they were satisfied (78%) with their current level of sexual intimacy (females: 77.8% versus males: 78.3%, *p* = 0.94), although half of all participants (58%) reported a lower sexual libido (sexual desire) after cancer (females: 60 % versus males: 55%, *p* = 0.54) and fatigue or lack of energy (62.7%) (females: 64.4% versus males: 60%, *p* = 0.58). Moreover, 60.7% of participants expressed feeling insecure regarding their ability to satisfy their partner after cancer (females: 64.4% versus males: 55%, *p* = 0.25).

**Fig. 2:**
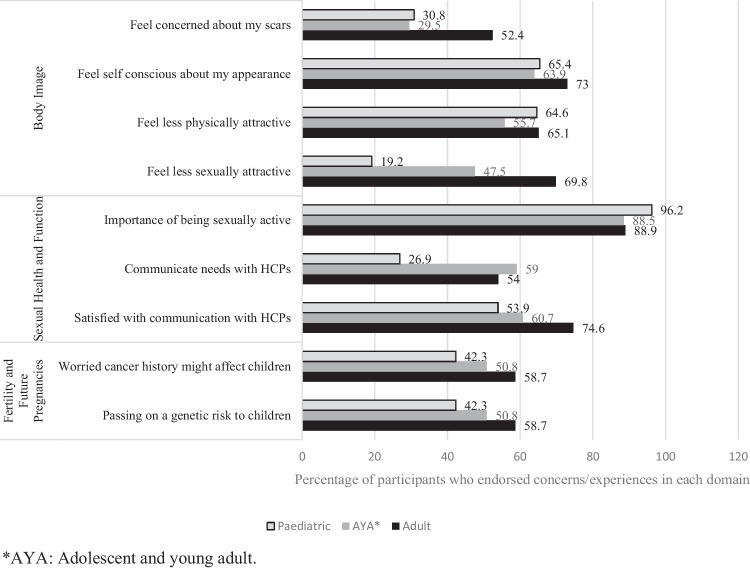
Sexual and reproductive concerns and experiences of cancer survivors (> 18 to ˂ 45 years) (*n* = 150)

Of the 137 participants who identified as being sexually active, 68% of participants (paediatric cancer survivors: 13.7%, AYA cancer survivors: 40.5% and adult cancer survivors: 45.8%) reported at least one concern about their sexual health and function, which had been affected after cancer treatment (Fig. 2). The internal consistency of the SHQ in this cohort was good, with a Cronbach’s alpha of 0.78.

Most participants (88.7%) indicated that they enjoyed engaging in sexual intimacy (females: 83.3% versus males: 96.7%, *p* = 0.01) and were satisfied with their ability to reach an orgasm (90.7%) (females: 85.6% versus males: 98.3%, *p* = 0.01). Less than half of all participants (42.7%) felt worried that they may experience some level of pain during sexual relations (females: 50% versus males: 31.7%, *p* = 0.03).

The validated EORTC sexual health tool included specific questions about communication but did not specify the type of HCP involved in communication or the type of communication. Half of all the participants (51.3%) reported having received some level of communication with an HCP about sexual health issues and reproductive concerns after cancer (females: 52.2% versus males: 50%, *p* = 0.79), with 65.3% of the participants reporting that they had engaged in discussions with their partner around their sexual health concerns either before or after cancer treatment (females: 63.3% versus males: 68.3%, *p* = 0.53).

### Reproductive preventative health care and contraception

Among participants that were sexually active, most reported not currently using any contraception (females: 61.1% versus males: 71.7%, *p* = 0.18). Slightly more than half of all female participants (60%) reported having had the human papillomavirus (HPV) vaccination and 60% of sexually active women reported that they had a cervical screening test.

### *Body Image Scale* [15]

Half of all participants (50.1%) endorsed at least one body image concern, with greater concerns in relation to the BIS reported by participants diagnosed with cancer as an adult patient (47.9%), followed by AYA (39.8%) and paediatric (12.3%) patients. Cronbach’s alpha in this sample was 0.88, indicating that the BIS was internally consistent.

In multivariate regression analysis, survivors tended to report feeling self-conscious about their appearance after cancer if they were female (*OR* = 4.10, 95% *CI* = 1.97–8.42, *p* < 0.001), had a partner at the time of completing the RS-PROM (*OR* = 2.51, 95% *CI* = 1.18–5.37, *p* = 0.02) and were never pregnant (*OR* = 2.16, 95% *CI* = 1.08–4.32, *p* = 0.03). No other demographic (e.g. age group at cancer diagnosis) or clinical factors (e.g. diagnosed with either a blood cancer or solid tumour and having had FP prior to cancer) were associated with this outcome.

Survivors who felt less physically attractive after their cancer treatment were more likely to be female (*OR* = 3.83, 95% *CI* = 1.84–7.95, *p* < 0.001), diagnosed with cancer as an AYA (*OR* = 4.01, 95% *CI* = 1.42–11.33, *p* = 0.01) or adult (*OR* = 4.70, 95% *CI* = 1.69–13.09, *p* = 0.003) patient and had a previous solid tumour, compared to haematological diagnosis (*OR* = 2.8, 95% *CI* = 1.36–5.75, *p* = 0.01). No other demographic (relationship status) treatment or clinical factors (e.g. had FP prior to cancer and ever been pregnant) were associated with feeling less physically attractive after cancer treatment.

Survivors reported feeling less sexually attractive as a consequence of disease or treatment if they were female (*OR* = 2.39, 95% *CI* = 1.13–5.04, *p* = 0.02), were in a relationship at the time of completing the RS-PROM (*OR* = 3.28, 95% *CI* = 1.51–7.14, *p* = 0.003), diagnosed with cancer as either an AYA (*OR* = 6.44, 95% *CI* = 1.99–20.88, *p* = 0.002) or adult (*OR* = 11.99, 95% *CI* = 3.72–30.61, *p* < 0.001) patient, and had undergone FP prior to starting cancer treatment (*OR* = 2.21, 95% *CI* = 1.13–4.30, *p* = 0.02).

Participants were also more inclined to report feeling dissatisfied with the physical appearance of a scar(s) following cancer treatment if they were female (*OR* = 2.36, 95% *CI* = 1.13–4.91, *p* = 0.02) and had been diagnosed with a solid tumour (*OR* = 5.37, 95% *CI* = 2.28–12.61, *p* < 0.001) or as an adult (*OR* = 2.76, 95% *CI* = 1.01-7.52, *p* = 0.05).

### Fertility concerns following cancer and future pregnancies

There were 36.1% of participants (14.5%, 40.8% and 44.8%; paediatric, AYA and adult cancer survivors, respectively) who reported having had at least one concern regarding their fertility or future pregnancies after cancer. The internal consistency of items included in this questionnaire in this cohort was good, with a Cronbach’s alpha of 0.88.

Half of all participants (54.7%) reported having had FP prior to starting cancer treatment; with significantly more male than female participants reported having undergone a FP procedure prior to cancer treatment (females: 42.4% versus males: 74.2%, *p* < 0.001) (paediatric: 11.5%, AYA: 65.6% and adult: 61.9% patients).

Most participants (64.7%) indicated that they were afraid that they would not be able to have any (more) children after cancer (females: 70% versus males: 56.7%, *p* = 0.09) with 60.7% of participants reporting concerns around not being able to achieve pregnancy in the survivorship period (females: 65.6% versus males: 5.3%, *p* = 0.13). Less than half of participants (33.3%) feared that they may not live long enough to take care of their child(ren) (females: 34.4% versus males: 31.7%, *p* = 0.72). Females compared with male participants more commonly stated that they were afraid that their potential offspring may have a higher chance of getting cancer (females: 40.0% versus males: 33.3% *p* = 0.04). When the same participants were asked whether they were concerned that they may not be able to have any (more) children we had similar results to being afraid of not having any (more) children (67.4% female, 60.3% male, *p* < 0.001) However, in multivariate analysis, male participants were more likely to feel concerned about how their family history might affect their future children’s health (*OR* = 2.62, 95% *CI* = 1.31–5.24, *p* = 0.01) and were more likely to express concerns about passing on genetic risk for cancer to their potential offspring (*OR* = 2.62, 95% *CI* = 1.31–5.24, *p* = 0.01), compared with female survivors. No other demographic (partner status, age group at cancer diagnosis) or clinical factors (cancer type, having FP prior to cancer treatment and, having had a previous pregnancy) were significantly associated.

Among participants that reported being in a relationship, few (17.3%) felt worried about telling their (potential) spouse/partner that they may be unable to have children in the future (females: 17.8% versus males: 16.7%, *p* = 0.86). 78% had actively initiated discussions with their spouse/partner about potential infertility and being unable to have children in the future prior to starting cancer treatment (females: 77.8% versus males: 78.3%, *p* = 0.94). There were 22.7% of participants who reported trying to get pregnant after cancer, and significantly more male than female participants had had achieved conception after cancer (females: 11.1% versus males: 40.0%, *p* < 0.001).

## Discussion

This is the first RS-PROM study to evaluate cancer survivors’ sexual and reproductive complications and concerns.

Only 60% of female cancer survivors reported having had a cervical screening test, indicating that preventative reproductive advice and education around this procedure to test for cervical cancer, in this cohort, is low, in keeping with the published literature [8]. Furthermore, uptake of the HPV vaccine was also low among female cancer survivors (60%), and although we did not collect data to support this outcome, this result may indicate that patients were in the hospital when the HPV vaccine was rolled out to schools or parents may have been concerned about their children receiving the vaccine. However, the literature reports the importance of administering the HPV vaccine to cancer survivors, as they are considered to be a vulnerable population at higher risk for HPV-related complications post-cancer [21]. A study conducted by Klosky et al. [21] reported that female bone marrow transplant recipients were at increased risk for both cervical dysplasia and cervical cancer. Furthermore, female cancer survivors who experience chronic graft versus host disease following a transplant and receive ongoing systematic immunosuppressive therapy for more than 3 years following transplant, are at higher risk for developing cervical cancer and other cervical abnormalities [22, 23].

More females compared with male participants had undergone hormone investigations (females: 38.9% versus 16.7%) and were on hormone therapy after cancer treatment (females: 34.4% versus males: 8.3%). Female participants were more likely to have undergone a bone density scan (females: 35.6% versus males: 11.7%) and tended to report higher rates of osteoporosis, brittle, weak or fragile bones (females: 12.2% versus males: 3.3%), but with no difference in fracture rate (females: 15.6% versus males: 10%). Cancer therapeutic regimens with chemotherapeutics, corticosteroids, aromatase inhibitors and androgen deprivation have shown to be significantly associated with fractures and bone loss which can negatively impact the skeletal health of a cancer survivor [24]. Cancer patients that are diagnosed with osteoporosis or osteopenia, are at higher risk of fractures than patients in the general population, which can negatively affect their quality of life^[ 25]^. Moreover, women and men who initiate hormone therapy (aromatase inhibitor and androgen deprivation therapy) should be offered discussions and counselling, regarding the adverse late effects of certain cancer therapies on bone health, both before starting cancer treatment and in the survivorship period [24]. Hence, a comprehensive bone therapy management plan should be incorporated into cancer patients’ treatment plans [24].

Cancer survivors frequently expressed concerns regarding their body image, including self-consciousness about their body image and feeling less attractive after cancer, more frequently expressed by female survivors than male survivors. The literature reports that these feelings are not uncommon among adolescents and adults in the general population, where the emphasis on body image and appearance is often negatively influenced by culture and social media [26]. Additionally, survivors reported feeling dissatisfied with their physical appearance particularly in relation to physical scarring (e.g. women who may undergo a mastectomy as part of breast cancer treatment), a prosthesis (e.g. following treatment for a sarcoma) weight loss or gain and hair loss [26–29]. Poor body image can have a negative effect on a cancer survivor’s quality of life, further creating feelings of unattractiveness and unworthiness, which can hinder both social and intimate relationships [7, 26].

Interestingly, few participants (22.7%) reported planning for a family in the survivorship period, and the reason for this might be that participants had already completed their families before starting treatment. There are also a number of other reasons patients may defer family planning after cancer, and these could include fears associated with relapse from their cancer, relationship status, financial constraints and many other medical and social factors [4, 30–32]. Moreover, male survivors expressed increased fears around the effect of their family history of cancer on future offspring’s health. Our findings are similar to those detailed in the literature, highlighting that cancer patients can experience psychological distress around issues associated with their potential offspring’s health being impacted by their cancer diagnosis [33, 34]. Further concerns have also been reported in relation to becoming a parent after cancer, more specifically around a cancer relapse as a result of pregnancy and not being able to look after a child or being at risk for higher obstetric and perinatal complications following cancer therapies (radiation and chemotherapy with alkylating agents) [35–37].

A recent study by Anazodo et al. [8] revealed that only 5% of consultations in the survivorship period had been documented in the patient’s notes regarding HCPs’ discussions with survivors around reproductive themes. Poor documentation of reproductive discussions and counselling between HCPs and patients around themes such as pubertal development (precocious puberty, delayed or absent pubertal development) [8, 42, 43]; menstrual dysfunction [8, 44]; endocrine complications [8, 45–47]; sexual dysfunction disorders [1, 8, 48]; infertility [8, 49]; and obstetric and perinatal complications [8, 50–52] limits collaboration between healthcare professionals outside of a survivorship clinic. HCPs may not have access to details about a patient’s cancer diagnosis or treatment, reproductive risks and fertility preservation details [53]. Hence, efficacious oncofertility care must incorporate expertise across several different health disciplines, as well as promote collaboration with multi-disciplinary teams of HCPs who provide paediatric, AYA and adult cancer care [8, 49].

The RS-PROM is a novel assessment tool that will allow patients to detail their own reproductive symptoms and concerns which may assist clinicians with a consultation in relation to a survivor’s sexual and reproductive concerns and needs. The RS-PROM may be useful in providing direct feedback to the clinician before a scheduled appointment to facilitate patient-focused discussions during the consultation. The RS-PROM can be utilised alongside existing patient-centred decision-making methods such as history taking, clinical examination and investigations to improve patient-clinician interaction.

## Strengths and limitations

### Strengths

This study had a relatively high participation rate [54], indicating the importance of reproductive healthcare to cancer survivors. The RS-PROM highlights cancer survivors’ sexual and reproductive concerns that survivors may be hesitant to address with a HCP [8]. The use of the RS-PROM at survivorship clinic consultations may help to identify the psychological reproductive needs and concerns of cancer survivors, thereby improving HCPs’ approaches to discussion and models of reproductive care.

### Limitations

Each of the three age cohorts included in this study was small, and patients had a range of different cancer types. The follow-up period for the cohort from diagnosis to completion of the RS-PROM was also large. Hence, there may be inherent biases in relation to patients who completed the RS-PROM in this study setting including potential recall bias. Also, paediatric survivors with a previous diagnosis of leukaemia were overrepresented in this cohort, which may have resulted in an underrepresentation of experiences associated with survivors of other paediatric cancers. Given that not all cancer services have an integrated cancer survivorship clinic, the findings presented may not be representative or generalisable to other cancer survivorship cohorts, particularly in less resourced settings. Moreover, the RS-PROM was piloted in English; therefore further research is required to understand the views of a culturally and linguistically diverse cohort of survivors. Consideration should also be given to piloting the RS-PROM in different clinical settings [55] at defined time intervals and with the use of telehealth platforms.

## Conclusion

The RS-PROM is a useful tool for identifying multiple reproductive complications and concerns for cancer patients in the survivorship period. Cancer survivors indicated concerns regarding their body image and felt self-conscious about their cancer. These concerns can have a significant impact on the quality of life of a survivor and can impact both the patient and their partner. Current models of survivorship care do not routinely provide sexual and reproductive health care in survivorship clinical settings, and many patients may not feel comfortable addressing these issues without a support framework. It may be possible to bridge the gap through the use of the RS-PROM so that the patient has an opportunity to discuss these issues with a HCP. The study’s findings may enhance patient and clinician interactions around sensitive topics, which has wider implications for guiding health care policy [12].

### Supplementary Information

Below is the link to the electronic supplementary material.

Supplementary file1 (PDF 236 KB)

Supplementary file2 (PDF 212 KB)

Supplementary file3 (XLSX 33 KB)

## Data Availability

The datasets generated during and/or analysed during the current study are available in the PROM repository.
